# Calmodulin‐mediated events during the life cycle of the amoebozoan *Dictyostelium discoideum*

**DOI:** 10.1111/brv.12573

**Published:** 2019-11-26

**Authors:** Danton H. O'Day, Sabateeshan Mathavarajah, Michael A. Myre, Robert J. Huber

**Affiliations:** ^1^ Cell and Systems Biology University of Toronto Toronto Ontario M5S 3G5 Canada; ^2^ Department of Biology University of Toronto Mississauga Mississauga Ontario L5L 1C6 Canada; ^3^ Department of Pathology, Faculty of Medicine Dalhousie University Halifax Nova Scotia B3H 4R2 Canada; ^4^ Department of Biological Sciences, Kennedy College of Sciences University of Massachusetts Lowell Lowell Massachusetts 01854 USA; ^5^ Department of Biology Trent University Peterborough Ontario K9L 0G2 Canada

**Keywords:** calcium signalling, calmodulin, asexual development, *Dictyostelium discoideum*, mitosis, osmoregulation, chemotaxis, cell differentiation, morphogenesis, extracellular matrix

## Abstract

This review focusses on the functions of intracellular and extracellular calmodulin, its target proteins and their binding proteins during the asexual life cycle of *Dictyostelium discoideum*. Calmodulin is a primary regulatory protein of calcium signal transduction that functions throughout all stages. During growth, it mediates autophagy, the cell cycle, folic acid chemotaxis, phagocytosis, and other functions. During mitosis, specific calmodulin‐binding proteins translocate to alternative locations. Translocation of at least one cell adhesion protein is calmodulin dependent. When starved, cells undergo calmodulin‐dependent chemotaxis to cyclic AMP generating a multicellular pseudoplasmodium. Calmodulin‐dependent signalling within the slug sets up a defined pattern and polarity that sets the stage for the final events of morphogenesis and cell differentiation. Transected slugs undergo calmodulin‐dependent transdifferentiation to re‐establish the disrupted pattern and polarity. Calmodulin function is critical for stalk cell differentiation but also functions in spore formation, events that begin in the pseudoplasmodium. The asexual life cycle restarts with the calmodulin‐dependent germination of spores. Specific calmodulin‐binding proteins as well as some of their binding partners have been linked to each of these events. The functions of extracellular calmodulin during growth and development are also discussed. This overview brings to the forefront the central role of calmodulin, working through its numerous binding proteins, as a primary downstream regulator of the critical calcium signalling pathways that have been well established in this model eukaryote. This is the first time the function of calmodulin and its target proteins have been documented through the complete life cycle of any eukaryote.

## I. INTRODUCTION

The eukaryotic social amoebozoan *Dictyostelium discoideum* has been a popular research organism for more than 80 years. Its original appeal was based on its lifestyle transition from single cell growth to multicellular asexual development characterized by chemotaxis, morphogenesis and cell differentiation. There are multiple reasons for this popularity: a well‐defined, rapid 24‐h multicellular life cycle; a haploid, sequenced genome; the ease of generating conditional and knockout mutants *via* diverse techniques; and the availability of multiple curated resources (e.g. strains, antibodies, data analysis programs) available through http://dictybase.org. Initially recognized for studies on fundamental biological processes, more recently it has been used a biomedical model for research into autophagy, cell stress, extracellular vesicles, osmoregulation, wound healing, neurodegenerative diseases, and mitochondrial diseases, among others (e.g. Annesley & Fisher, Annesley & Fisher, 2001; Huber, 2016; Mathavarajah, O'Day, & Huber, 2018b; Dunn *et al.,* 2018; Myre, Huber, & O'Day, 2018; Tatischeff, 2019).

**Figure 1:**
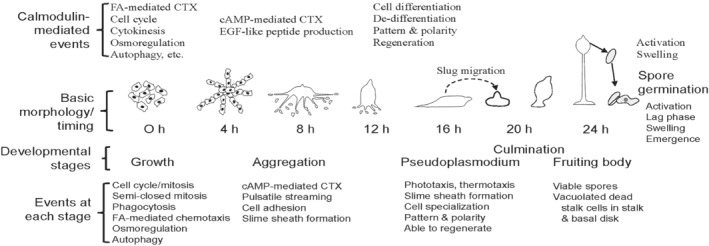
Calmodulin functions during asexual development in *Dictyostelium discoideum*. cAMP, cyclic AMP; CTX, chemotaxis; EGF, epidermal growth factor; FA, folic acid.

The asexual life cycle of this eukaryotic microbe is summarized in Fig. 1. After feeding on bacteria, where folic acid‐mediated chemotaxis aids in food detection and ingestion, starved cells switch to cyclic AMP (cAMP)‐mediated chemotaxis that guides their pulsatile migration towards aggregation centres. The aggregates grow upwards until they topple over as multicellular pseudoplasmodia that, depending on environmental conditions, can either culminate directly or migrate for extended periods in response to heat, light and other factors. The cellular nature of the amoebozoan pseudoplasmodia differ from the multinucleate plasmodia of mycomycetes (true slime molds). Within these pseudoplasmodia, also commonly referred to as ‘slugs’, cell differentiation begins with a well‐defined pattern and polarity. The pre‐spore cells reside in the hind two‐thirds to three‐quarters of the slug while the anterior one‐third to one‐quarter of the slug contains cells destined to differentiate into stalk cells. The pre‐stalk cohort is heterogeneous consisting of A, AB, and O cells, each with distinct fates and patterns of gene expression. Anterior‐like cells (ALCs) distribute throughout the pre‐spore region where they serve as a source of renewal for the anterior pre‐stalk cells. Pseudoplasmodia can regenerate if disrupted or transected, as detailed below.

During culmination, cells in the pre‐stalk region flow like a reverse fountain through the pre‐spore region where cells from three groups of pre‐stalk cells (i.e. AB, B, ALCs) will form the basal disc that supports the growing stalk. Differentiation of other prestalk cells (i.e. A, AB, O) causes the stalk to elongate lifting the differentiating spore mass off the substratum ultimately to reside at the tip of the elongated stalk. During these events, the stalk cells die and become the supporting structure for the viable spores that will start the next generation. In the spore mass, the spores of *Dictyostelium* float freely in an aqueous, protein‐rich extracellular fluid. Since there is no surrounding wall around this spore droplet, it cannot be considered a sorus or sporangium, terms well established for plants and fungi.

Calcium (Ca^2+^) function is universal in eukaryotes and evolutionary research indicates that the first signal transduction systems utilized this ubiquitous divalent cation (e.g. Peterson, Michalak, & Verkhratsky, 2015; Plattner & Verkhratsky, 2015). The transition from single‐celled eukaryotes to metazoans was accompanied by increasing complexity in how Ca^2+^ is utilized for signal transduction. This includes the evolution of distinct protein machinery in protozoans, such as the emergence of store‐operated Ca^2+^ entry, prior to the appearance of animals (Collins & Meyer, 2011). Undergoing developmental phase transitions from single cells to a *bona fide* multicellular tissue during development puts *D. discoideum* in a unique position to provide critical insight into the evolution of calcium signal transduction. During animal development, Ca^2+^ functions in multiple events including cell death, differentiation, division and motility. It is involved in biomembrane fusion (e.g. fertilization, secretion, endocytosis, etc.), morphogenesis and the specialization of bone, heart and neurons, to name a few cell types (e.g. Webb & Miller, 2003; Zhou *et al.,* 2011).

## II. CALCIUM–CALMODULIN SIGNALLING IN *DICTYOSTELIUM DISCOIDEUM*

As in all other eukaryotes, Ca^2+^ signalling is central to the survival and physiological function of *D. discoideum*. Ca^2+^‐mediated events are, in turn, dependent on the activation of Ca^2+^‐binding proteins (CBPs) of which calmodulin (CaM) is the most predominant. *D. discoideum* CaM [152 amino acids (aas), 17 kDa] is encoded by the *calA* gene (http://dictybase.org/gene/DDB_G0279407). It is expressed throughout growth, steadily decreasing in expression throughout asexual development (Van Driessche *et al.,* 2002). It contains four Ca^2+^‐binding EF hands (a helix–loop–helix structural motif) and is over 90% identical to mammalian CaM, being able to fully activate mammalian CaM‐binding proteins (CaMBPs) such as bovine brain cyclic nucleotide phosphodiesterase and erythrocyte Ca^2+^/Mg^2+^‐ATPase (Bazari & Clarke, 1981). It differs in the lack of Lys‐118 trimethylation, a mechanism of post‐translational regulation. It is the primary CBP involved in activating a diversity of downstream proteins in its Ca^2+^‐free (apo‐CaM) or Ca^2+^‐bound (Ca‐CaM) states as detailed herein. Intracellularly, CaM is found in the nucleus, nucleolus and contractile vacuole (CV) system as well as various regions throughout the cytoplasm (e.g. cytoskeleton). CaM is also constitutively secreted where it functions extracellularly to inhibit cell proliferation and increase cAMP‐mediated chemotaxis. (O'Day, Huber, & Suarez, 2012; see Section V).

The related *calB* encodes a Ca^2+^‐binding, CaM‐like protein consisting of 149 aas of 16.8 kDa. While its lowest level of expression occurs during growth, *calB* is differentially expressed during asexual development (Rosel *et al.,* 2000). During aggregation through to the pseudoplasmodium stage it increases in expression and is expressed at the highest level in spores. CalB shows about 50% similarity to other CaMs but contains only two Ca^2+^‐binding EF hands. However, the precise function of CalB remains to be studied. Another CaM‐related protein, Myosin‐ID light chain (MlcD; 147 aas, 16.5 kDa) is a low‐affinity Ca^2+^‐binding protein that operates as the light chain for myosin‐D (MyoD) but not MyoB or MyoC (De La Roche, Lee, & Coté, 2003).

In eukaryotes, CaM is a highly conserved Ca^2+^‐sensor and effector that mediates a diversity of biological processes through its regulation of hundreds of target CaMBPs (Chin & Means, 2000). In the absence or presence of Ca^2+^, it regulates the activity of ion channels, enzymes, heterotrimeric G‐proteins, transcription factors, transport systems and other critical proteins. As the primary mediator of intracellular Ca^2+^ signal transduction, it oversees the operation of a diversity of growth factors, hormones and neurotransmitters. As such, it carries out critical operations in a diversity of biological processes (e.g. apoptosis, autophagy, cell differentiation, cell division, morphogenesis, osmoregulation, etc.) and pathophysiological states (e.g. cancer, neurodegeneration, heart arrythmias, etc.) (Berchtold & Villalobo, 2014; Villalobo, 2018; Urrutia *et al.,* 2019). The importance of *D. discoideum* as a model system to investigate fundamental biological processes and disease states is gaining increased attention (Annesley & Fisher, 2001; Huber, 2016; Dunn *et al.,* 2018; Myre *et al.,* 2018; Tatischeff, 2019). Here we show the critical role of CaM and its CaMBPs throughout the full developmental cycle of *D. discoideum* to emphasize its importance for continued study of specific cellular events and disease processes. Considering the diversity and central importance of CaM function, understanding how it can modulate such a diversity of precise events, often in the same cell, in different physiological and developmental events, argues that this area of research has the potential to yield extraordinary insight and pharmaceutical targets.

Just over a decade ago, Catalano & O'Day (2008) catalogued the CaMBPs of *D. discoideum*, their specific functions and their CaM‐binding domains (CaMBDs). CaMBDs are unique since they do not involve a precise sequence of specific amino acids (Hoeflich & Ikura, 2002; Berchtold & Villalobo, 2014). Calcium‐independent CaM‐binding occurs through IQ [(FILV)Qxxx(RK)Gxxx(RK)xx(FILVWY)] or IQ‐like [(FILV)Qxxx(RK)Gxxxxxxxx] motifs. Calcium‐dependent CaMBDs employ sequences that fall into two categories: canonical and non‐canonical binding motifs. Canonical CaMBDs comprise a diverse collection that are defined by the positions of hydrophobic residues [e.g. 1–5‐10 motif: (FILVW)xxxx(FAILVW)xxxx(FILVW); 1–5–8‐14 motif: (FILVW)xxx(FAILVW)xx(FAILVW)xxxxx(FILVW)]. Non‐canonical binding does not appear to involve definable amino acid patterns.

*D. discoideum* CaMBPs with identified binding domains include calcineurin (CanA), cysteine‐rich protein A (CyrA), nucleomorphin (NumA1) and phosphoglycerate kinase (PgkA). CaMBPs where the CaM binding was experimentally verified and putative CaMBDs were identified but not experimentally proven include: calmodulin‐binding protein 46 (CmbB), Ras GTPase‐activating‐like protein (RgaA), histone H1 (H1), ribosomal subunit protein L19 (Rpl19), thymidine kinase (ThyB) and Von Willebrand factor kinase A (VwkA). In addition to covering all the newly identified CaMBPs and their functions, the attributes and roles of proteins previously reviewed by Catalano & O'Day (2008) are updated and linked to specific events during the *D. discoideum* life cycle. Other CaMBPs that were reviewed by Catalano & O'Day (2008) but which since have not been detailed further are not covered here: PgkA, histone H1, Rpl19 and ThyB.

Studying a group of proteins that are controlled by a common regulator can lead to new insights that may not arise when studying a single protein. The study of CaMBPs in *D. discoideum* has led to the discovery of many proteins that at first seemed unrelated in localization and function but, with continued work, revealed a series of previously unexpected interplays. By focussing for the first time on CaM during a complete eukaryotic life cycle, this review provides greater insight into the importance of this regulatory protein in this model eukaryotic amoebozoan while simultaneously revealing specific translational insight into various fundamental cellular events.

## III. CELL MOTILITY AND CHEMOTAXIS DURING GROWTH

During growth, *D. discoideum* amoebae feed on bacteria and other microbes. Their ability to locate their food supply is enhanced through their positive chemotactic response to folic acid secreted by bacteria (Van Driel, 1981). Recently, Pan *et al*. (2016) revealed that folic acid binds to a receptor that not only allows cells to locate bacteria but also to ingest them. Schlatterer & Malchow (1993) demonstrated the importance of Ca^2+^ signalling in chemotaxis to folic acid. Subsequent research showed that a diversity of CaM‐dependent effectors is involved in the motility of *D. discoideum* cells: RgaA, myosin light chain kinase (MlkA), myosin heavy chain kinase (MhkA), and many myosin I isoforms (reviewed in Catalano & O'Day, 2008). Thus, it came as no surprise when chemotaxis to folic acid was shown to be CaM‐dependent (Gauthier & O'Day, Gauthier & O'Day, 2001). Profiling revealed the presence of dozens of Ca^2+^‐dependent CaMBPs in cytosolic and nuclear fractions plus a small population of membrane‐localized proteins in growth‐phase cells (Gauthier & O'Day, Gauthier & O'Day, 2001). Starvation and refeeding experiments linked a handful of these to events including chemotaxis, but most of the proteins remain uncharacterized.

Other experiments revealed the CaM‐dependent serine/threonine phosphorylation of proteins linked to folic acid chemotaxis, thus reinforcing the role of CaM in this process (Gauthier & O'Day, Gauthier & O'Day, 2001). Since CanA, the sole CaM‐dependent protein phosphatase, dephosphorylates both serine and threonine residues, it was suggested that it could function during folic acid‐mediated chemotaxis. CanA was the first CaMBP to be characterized in *D. discoideum* (Catalano & O'Day, 2008; Damman *et al*., Dammann *et al.,* 1996; Thewes *et al.,* 2016). However, despite its role in a diversity of chemotactic events in other species, investigations into the role of CanA suggest it is not linked to folic acid‐mediated chemotaxis. Instead, it is critical to numerous other basic cellular processes essential for growth and, as detailed later, asexual development (Kobel‐Höller *et al.,* 2018). However, CanA has been implicated in cAMP‐mediated chemotaxis, fitting with the results of Gauthier & O'Day (Gauthier & O'Day, 2001) who showed significant differences in the CaMBP profiles of cells capable of folic acid‐mediated chemotaxis *versus* those undergoing cAMP‐mediated chemotaxis.

Various myosins and kinases function in motility and chemotaxis. Myosin light chain C (MlcC), a regulator of myosin‐1C (Myo1C), is one of the best characterized CaMBPs in *D. discoideum* (e.g. Langelaan *et al.,* 2016). It binds to apo‐CaM *via* the Ca^2+^‐independent IQ motif (summarized in Catalano & O'Day, 2008). During cell motility, Myo1C localizes to the cell cortex where it interacts with actin to generate cortical tension as part of the events leading to the extension and retraction of the cell membrane (Langelaan *et al.,* 2016). The CaM‐mediated regulation of myosin‐based contraction involves MhkA, myosin heavy chain kinase B (MhkB) and the myosin I heavy chain kinase P21‐activated protein kinase (PakB), each of which has been shown to contain presumptive CaMBDs (see Table 1 in Catalano & O'Day, 2008). Other functions for cortical Myo1C during the growth phase include pinocytosis (Langelaan *et al.,* 2016).

Until recently, the receptor for folic acid remained elusive. Using a novel quantitative phosphoproteomic technique, Pan *et al*. (2016) identified fAR1, a G‐protein coupled receptor (GPCR) that is essential for folic acid‐mediated signal transduction. In addition, the receptor is essential for both chemotaxis and bacterial phagocytosis. Because of the importance of CaM in folic acid‐mediated chemotaxis and since many G‐protein‐coupled receptors are CaMBPs, a scan of fAR1 was performed to assess the presence of potential CaMBDs (Yap *et al.,* 2000; Hoeflich & Ikura, 2002). A CaM‐binding region (611CVLIIFGAKFWKIYKPVEDD630) was identified within which six distinct canonical binding motifs were present, strongly supporting fAR1 as a CaMBP (D.H. O'Day, unpublished data).

## IV. GROWTH PHASE AND SEMI‐CLOSED MITOSIS

While researchers have been unable to synchronize the cell cycle of *D. discoideum* sufficiently, this has not limited its value in the study of this critical process, not only as a biomedical research model but also for elucidation of related evolutionary events. The presence of CaM in the nucleus of this microbe has been experimentally verified (Huber, Catalano, & O'Day, 2013). The cell cycle includes semi‐closed mitosis wherein the nuclear envelope does not break down but allows the regulated nucleocytoplasmic transport of various CaMBPs and some of their binding partners (O'Day & Budniak, 2015). For example, cyclin‐dependent kinase 5 (Cdk5) is a nuclear CaMBP that progressively translocates from the nucleoplasm to the cytoplasm during mitosis, re‐entering the nucleus during cytokinesis (Huber *et al.,* 2013). *D. discoideum* Cdk5 is involved in multiple events during growth and development including cell differentiation, endocytosis, phagocytosis, proliferation and secretion (Sharma *et al.,* 2002; Huber & O'Day, 2011b, 2012b). During interphase, Cdk5 binds to the metalloproteinase puromycin‐sensitive aminopeptidase A (PsaA) in the nucleus in a Ca^2+^/CaM‐dependent manner. PsaA also possesses CaMBDs, and immunoprecipitation experiments with nuclear extracts co‐precipitate CaM, Cdk5 and PsaA (Huber *et al.,* 2013).

In keeping with its role in other organisms, the significance of PsaA in the cell cycle was suggested initially by its nuclear co‐localization and co‐immunoprecipitation with NumA1 (Catalano, Poloz, & O'Day, 2011). NumA1 is a nucleolar/nucleoplasmic, BRCA1 C‐terminal (BRCT) domain‐containing CaMBP involved in mitosis in *D. discoideum* (Myre & O'Day, 2002, 2004a, 2004b, 2005). Showing a predominantly nuclear localization during interphase, PsaA translocates to the cytoplasm during mitosis (Catalano *et al.,* 2011). The study also identified the first nuclear localization sequence (680RKRF683) for *D. discoideum*. Despite the presence of a putative CaMBD and binding to two CaMBPs, NumA and Cdk5, neither Ca^2+^ nor CaM are required for the nuclear localization of PsaA. More about the role of this enzyme in multicellular development and differentiation is discussed below (see Section XIV).

In addition to containing experimentally verified CaM‐binding and BRCT domains, as well as nuclear (NLS) and nucleolar localization sequences (NoLS), the nuclear/nucleolar protein NumA also contains a DEED domain, an extensive palindromic stretch of repeating aspartic acid and glutamic acid residues (Myre & O'Day, 2002, 2004a, 2004b). The highly acidic DEED domain shares similarities with a sequence found in nucleoplasmin, a protein that binds to core histones where it is involved in chromatin condensation and DNA transcription (Onikubo *et al.,* 2015). Overexpression of NumA constructs lacking the DEED domain results in multinuclearity while not altering the nucleolar/nuclear localization of the protein (Myre & O'Day, 2002). It should be noted that during mitosis, as in other organisms, the nucleolus disappears during prophase, reappearing during telophase. Fitting with this, NumA localization becomes nucleoplasmic during prophase then it becomes predominantly cytoplasmic until nucleolar reorganization (O'Day, 2019). To understand how the DEED domain might mediate nuclear number, immunoprecipitation and yeast two‐hybrid studies revealed that Ca^2+^‐binding protein 4a (Cbp4a) bound to NumA *via* the DEED sequence (Myre & O'Day, 2004a). While these studies so far have failed to reveal the answer, they provide valuable insight into critical aspects of mitosis in this eukaryotic microbe. Unlike NumA, PsaA and Cdk5 that translocate from the nucleus to the cytoplasm during mitosis, Cbp4a localizes to the nucleoplasm after nucleolar dissolution where it organizes into multiple, discrete nucleoplasmic islands (Catalano & O'Day, 2013; O'Day, 2019). These translocations along with those of other proteins led to the realization that *D. discoideum* has a semi‐open mitosis as opposed to a closed mitosis as previously thought (O'Day & Budniak, 2015). Semi‐open mitosis refers to a form of mitosis in which the nuclear envelope undergoes fenestration to be partially but not completely disrupted. It is observed in certain fungal species such as *Ustilago maydis* and *Schizosaccharomyces japonicas* (Boettcher & Barral, 2013).

Less is known about CaMBPs involved in the final event of cell division: cytokinesis. However, the *Dictyostelium* WW domain‐containing protein A (DwwA) is a calcium‐independent, single IQ‐domain CaMBP that is essential for this event (Catalano & O'Day, 2008).

## V. EXTRACELLULAR CALMODULIN DURING GROWTH

Before solid experimental evidence was presented for the extracellular presence and function of CaM, several studies had suggested its presence in a diversity of plant and animal species. Although not noted in their publication, a scan of the data from a proteomics‐based analysis of secreted proteins during *D. discoideum* development indicated the presence of extracellular CaM (eCaM; Bakthavatsalam & Gomer, 2010). Also present were several CaMBPs including Cbp4a, PsaA, CmbB and CmbC. Suarez *et al*. (2011) subsequently revealed that eCaM was involved in the processing of the matricellular protein CyrA as discussed below (see Section XII). O'Day *et al*. (2012) followed up with a detailed study on eCaM showing that it is present at high and consistent levels throughout asexual development where it functions during at least three different events. It dose‐dependently inhibits cell proliferation during growth and enhances cAMP‐mediated chemotaxis during development. eCaM localizes to the extracellular matrix (ECM) where it is involved in processing CyrA to release fragments containing bioactive epidermal growth factor (EGF)‐like repeats (see Section XII). While the mechanism of eCaM secretion remains to be clarified, two primary routes seem reasonable. First, it could be translocated in association with one or more extracellular CaMBPs, such as CyrA, or it could be released during the expulsion phase of CV system function. The relationship between CaM and the CV system is detailed below (see Section VIII). Since CaM serves multiple functions intracellularly, it is likely that more extracellular functions will be revealed in future studies.

## VI. PHAGOCYTOSIS

*D. discoideum* is a professional phagocyte that has provided valuable insights into the early evolution of the immune system (Chen, Zhuchenko, & Kuspa, 2007; Dunn *et al.,* 2018). However, while Ca^2+^ signal transduction mediated by CaM is essential for phagocytosis in both animal, protozoa and plant cells, the importance of Ca^2+^/CaM signalling in this process in *D. discoideum* remains unstudied. As discussed above, Pan *et al*. (2016) identified fAR1, a folic acid G‐protein‐linked receptor that mediates both chemotaxis and phagocytosis which, as indicated above, is a potential CaMBP. They proposed that mammalian phagocytes might use the same system of chase (chemotaxis) and engulf (phagocytosis) that *D. discoideum* employs during bacterial feeding. The raw data on the genes linked to phagocytosis, based on transcriptional changes, from a study by Sillo *et al*. (2008) were analysed for the presence of CaMBP‐encoding genes, but only one was identified (CmbB). CmbB is almost entirely comprised of tandem IP22 (FNIP) repeats (O'Day *et al.,* 2006). It binds CaM *via* a CaMBD (179IPKSLRSLFLGKGYNQPLEF198) that contains canonical 1–10 and 1–15 binding motifs.

The highly conserved, multi‐functional protein nucleotide diphosphate kinase (NDPK) is an enigma. Its primary housekeeping function is to phosphorylate nucleotides, but it also is involved in a diversity of other processes that are typically unrelated to its enzymatic function. *D. discoideum* has three NDPK genes (mitochondrial *ndkM, cytosolic ndkC, and uncharacterized gene DDB_G0292928*) of which cytosolic nucleotide diphosphatase kinase C (*NdkC)* is the most predominant (Annesley *et al.,* 2011). *NdkC* functions to inhibit phagocytosis and macropinocytosis, and it co‐localizes with CaM at the CV system. It should be noted that the phagocytic data of Sillo *et al*. (2008) did not reveal *NdkC*. Citing unpublished data, Annesley *et al*. (2011) stated that *NdkC* did not co‐immunoprecipitate with CaM. While this suggests it is not a CaMBP, sequence analysis reveals that cytosolic *NdkC* contains a CaMBD (23GLVGEIIARYEKKGFVLVGLKQLV46) with six different canonical binding motifs (3 × 1–14, 2 × 1–16, 1 × 1–5‐10, 1 × 1–10; D.H. O'Day, unpublished data). Further to this, the authors did not provide details on the methodology or antibodies that were used to assesss an interaction between CaM and NdkC. This suggests that *NdkC* remains a potential CaMBP that requires further study.

## VII. AUTOPHAGY DURING GROWTH AND DEVELOPMENT

Autophagy is a critical metabolic pathway that is highly conserved in eukaryotes. The term autophagy describes a group of cellular pathways (including macro‐ and microautophagy) that oversee the elimination of intracellular material through lysosomal digestion. Seminal work in yeast established the genetics underlying autophagy (Levine & Klionsky, 2017). Many of the genes in yeast that regulate autophagy also function in *D. discoideum* (Calvo‐Garrido *et al.,* 2010). While autophagosomes in yeast originate from a concentrated cytoplasmic region, autophagy induction in *D. discoideum* resembles the stages in mammalian autophagy, where nascent autophagosomes originate from multiple cytoplasmic locations (Otto *et al.,* 2004; Tekinay *et al.,* 2006). The autophagy‐related 1 (Atg1/ULK) complex initiates autophagy in unikonts (which includes yeast, *Dictyostelium* and animal cells) but alternative strategies have also been suggested for non‐unikont parasites (such as *Trypanosoma*) (Földvári‐Nagy *et al.,* 2014). In *D. discoideum*, this complex is composed of Atg1, Atg13, Atg101 and DDB_G0285767 (ortholog of Atg11) (Mesquita *et al.,* 2015, 2017). By utilizing the Calmodulin Target Database, we found that each protein from the Atg1 complex in *D. discoideum* possesses putative CaMBDs (Yap *et al.,* 2000). One or more Ca^2+^‐dependent non‐canoncal and canonical CaMBDs were identified for Atg1 (non‐canonical _110_EKALYFMKQLAN_121_), Atg13 (1–10, 1–16, 104FYKNVIILIRTIYAML129), Atg101 (1–14 and 1–16, 72LTSIMKRKAKTAQISI86), and DDB_G0285767 (1–16, 172FFEYRENLIKTANKSF191; 1–14, 1–16, 1–5‐10, 240WYKHLKEEFSKVKLKV255). The potential for CaM to bind the Atg1 complex aligns with recent observations in *D. discoideum* where rapamycin (a drug which initiates autophagy) treatment increases intracellular Ca^2+^ levels (Swer, Lohia, & Saran, 2014).

During growth in nutrient‐rich media, many of the *D. discoideum* autophagy genes appear to be dispensable (Otto *et al.,* 2003, 2004). Known exceptions to this are *atg8a* and *atg9*, where their loss leads to reduced growth (Tung *et al.,* 2010; Messling *et al.,* 2017). Atg9 is found on distinct Golgi‐derived vesicles that are a source of autophagosomal membrane (they fuse to the autophagosomal outer membrane) and it is consequently involved in forming autophagosomes (Orsi *et al.,* 2012; Yamamoto *et al.,* 2012). It has been shown that the induction of autophagy by thapsigargin occurs through increased intracellular Ca^2+^ signalling (Luciani *et al.,* 2011; Marcassa *et al.,* 2017). The resulting Ca^2+^‐activated calpains mobilize Atg9‐positive vesicles for autophagosome formation in human cells. Early research had shown that calpains often target CaMBPs (Wang, Villalobo, & Roufogalis, 1989). A Calmodulin Target Database scan revealed a putative, unusually short CaMBD in *D. discoideum* Atg9 (221IANRIMRK228) suggesting the ortholog may also bind to CaM but *via* a non‐canonical motif (S. Mathavarajah, unpublished data). This idea is strengthened by the result that CaM co‐immunoprecipitated during a proteomic screen of Atg9 interactors in human cells (Kakuta *et al.,* 2017). These results link Ca^2+^ fluxes to the progression of autophagy and suggest a potential role for CaM in regulating autophagosome formation.

During starvation, increased intracellular Ca^2+^ is thought to be involved in the signalling cascade regulating the transition to multicellular development (Tanaka *et al.,* 1998). Similarly, nutrient depletion in mammalian cells causes a transient increase in intracellular Ca^2+^ levels leading to the initiation of autophagy (Høyer‐Hansen *et al.,* 2007). This signalling mechanism may also initiate developmental autophagy in *D. discoideum* (Otto *et al.,* 2003). The independent loss of *atg5*, *atg6*, *atg7*, *atg8a*, *atg9* or *atg101* leads to multi‐tipped aggregates (Otto *et al.,* 2004; Calvo‐Garrido *et al.,* 2010; Tung *et al.,* 2010; Messling *et al.,* 2017). Multi‐tipped aggregates are a characteristic phenotype of *D. discoideum* autophagy mutants, and this has been used to identify other autophagy‐regulating proteins in *D. discoideum* multiple tip mutations (e.g. TipC, TipD) (Muñoz‐Braceras, Calvo, & Escalante, 2015). The nature of this phenotype also suggests that autophagic machinery is involved in the differentiation of cell types (Mesquita *et al.,* 2017).

As discussed below (see Section XIV), Ca^2+^/CaM signalling is critical in stalk cell differentiation. It involves a process that resembles autophagic cell death (ACD). Work has shown that the induction of ACD in *D. discoideum* requires the Atg1 complex (Kosta *et al.,* 2004). Based on the putative CaMBDs of the Atg1 complex proteins and Atg9, we hypothesize that there is potential for CaM regulation of autophagy induction during ACD. In addition to this, ACD is regulated by the inositol 1,4,5‐trisphosphate receptor (encoded by *iplA*) that governs the efflux of Ca^2+^ from the endoplasmic reticulum (ER) (Lam *et al.,* 2008). IplA has been studied extensively as a CaMBP: it is inhibited by the actions of CaM at high Ca^2+^ concentrations but not at low Ca^2+^ levels (Kasri *et al.,* 2002). IplA also has a predicted CaMBD (1–10, 841VSKGRNYNGI850; 1–14, 850IRLVGQRITHKECL863) based on Calmodulin Target Database scanning and is likely regulated in a similar way (Yap *et al.,* 2000; D.H. O'Day & S. Mathavarajah, unpublished data). This indicates that in addition to stalk cell differentiation, ACD may be regulated by CaM through its interactions with IplA. This idea is taken further in a discussion of IplA and CaM in spore germination (see Section XVI).

## VIII. OSMOREGULATION AND VACUOLE FUNCTION

The CV system of *D. discoideum* and its relationship to those found in other organisms has been reviewed previously (Plattner, 2013). As a soil microbe, the osmoregulatory function of the *D. discoideum* CV system is critical for survival. Plattner (2013) summarized the importance of CaM in CV function. The CV system is bipartite consisting of a tubular spongiomal network that feeds into a bladder‐like vacuole from which a pore can form to release water from the distended vacuole (Nolta & Steck, 1994). Taking direction from previous work on the protozoan *Paramecium*, wherein CaM binds to the membranes of its well‐defined CV complex, Zhu & Clarke (1992) first showed that the less‐well‐defined CV system of *D. discoideum* also contained membrane‐bound CaM along with multiple, unidentified CaMBPs. A CaM‐binding unconventional myosin I isoform was one of these proteins. Subsequent work verified that MyoJ (a type V myosin) localizes with CaM in CV system bladders but it is likely that binding between the two is not essential for CaM localization or CV system function (Jung, Titus, & Hammer, 2009). It was also shown that CaM binds to a modifier of myosin II that localizes to the CV system, VwkA (a kinase that affects myosin II abundance and assembly). The autophosphorylation activity of VwkA is enhanced by CaM binding (Betapudi *et al.,* 2005). Taken together, CaM appears to have a role in regulating the cytoskeletal changes that occur in response to osmotic stress.

Zhu & Clarke (1992) also offered unpublished support for the presence of an essential vacuolar H^+^‐ATPase (V‐ATPase), a discovery supported by other research (Liu & Clarke, 1996; Plattner, 2013). Despite their early work on the importance of CaM to CV system function, the CaM binding of the V‐ATPase was not assessed. V‐ATPase is a multi‐subunit mechanical protein consisting of two subcomplexes: a water‐soluble V_1_ shaft and a membrane‐embedded V_0_ channel (Tirtom *et al.,* 2013). In *D. discoideum*, the 100 kDa V_0_ channel is encoded by the *vatM* gene. V‐ATPase is critical for the acidification of endosomal compartments and contributes to the membrane potential of the CV system (Clarke *et al.,* 2002; Grønlien *et al.,* 2002). This H + ‐ATPase was recently also shown to be localized to phagosomes, alluding to multiple functions within the cell (Clarke *et al.,* 2002). A Calmodulin Target Database scan of V‐ATPase subunit M (VatM) revealed a non‐canonical Ca^2+^‐dependent CaMBD motif (291DHKRQTLAGIV301) and one Ca^2+^‐independent IQ‐like motif (357IQLALRTATTRSGA360) suggesting that VatM may bind to CaM in both the presence and absence of Ca^2+^ (D.H. O'Day, unpublished data). This interaction is supported by work using CaM antagonists (W‐7 and W‐5) that reduce the ATP‐dependent acidification of acidic compartments (Malchow, Lusche, & Schlatterer, 2004). The highly potent W‐7 [N‐(6‐aminohexyl)‐5‐chloro‐1‐naphthalenesulfonamide] significantly reduced acidification, suggesting that the normal function of V‐ATPase requires CaM binding. Independent of acidification, CaM interaction with an ortholog of VatM in *Drosophila melanogaster* regulates the assembly of soluble N‐ethylmaleimide‐sensitive factor attachment protein receptors (SNAREs), that have been shown to facilitate the membrane‐to‐membrane contact required for CV system discharge in *Paramecium tetraurelia* (Schönemann *et al.,* 2013; Wang *et al.,* 2014). This suggests that the interaction of CaM with V‐ATPase subunits influences osmoregulation in a multi‐faceted manner in terms of CV system acidification (or membrane potential) and SNARE‐mediated CV system discharge.

Over 24 different proteins have been found to be associated with the CV system in *D. discoideum*, a few only transiently (Plattner, 2013). Of these and those subsequently identified, only a limited number have been identified as CaMBPs. The BEACH (Beige and Chediak‐Higashi) domain‐containing protein LvsA, a critical protein for the maintenance of vacuole integrity, requires CaM for its binding to CV system membranes (Malchow *et al.,* 2006). This is a reciprocal relationship between CaM and LvsA, as the loss of LvsA leads to the absence of CaM at CV system membranes (Gerald, Siano, & De Lozanne, 2002).

Cad1, a Ca^2+^‐dependent, homophilic cell‐to‐cell adhesion molecule that binds to and enters vacuoles, was identified and verified as a CaMBP by Sriskanthadevan *et al*. (2009). Produced as a soluble protein early in development, Cad1 is inserted into the plasma membrane through its association with the vacuoles of the CV system. Co‐immunoprecipitation analyses revealed that vacuole docking and uptake of Cad1 is Ca^2+^/CaM dependent and that both events are inhibited by CaM antagonists. Cad1 also has functions in cell sorting and morphogenesis as discussed below (see Section XIV).

The P‐type ATPase (PatA), a plasma membrane Ca^2+^‐ATPase‐type pump, is a CV system protein but was identified as lacking the conserved inhibitory CaMBD found in other species (Pittman, 2011). However, a binding site scan revealed one CaMBD in PatA (1–10, 1–14, 1064WQIVRQTHKKLVVINALKE1085) that contains multiple canonical binding motifs, a strong indicator of CaM binding. The importance of PatA and other Ca^2+^ pumps led to the discovery that the CV system is also critical for the regulation of Ca^2+^ levels during cAMP‐mediated chemotaxis, a subject that is discussed in Section IX (Malchow *et al.,* 2006).

Du *et al*. (2008) proposed a disgorgin‐based model for the regulation of CV system function that included the sequential functions of dajumin, Ras‐related protein Rab‐1A (Rab11A), Ras‐related protein Rab‐1C (Rab11C), drainin, Ras‐related protein Rab‐8A (Rab‐8A), disgorgin and BEACH domain‐containing protein lvsA (LvsA), but not other critical proteins such as PatA or VatM. More importantly, despite its central role in CV system structure and function, CaM was not included in the model. Various Rab family proteins either bind CaM directly or *via* their activating proteins (e.g. Zhu *et al.,* 2016). Sequence analysis of *D. discoideum* Rab11A and Rab11C revealed they both contain canonical Ca^2+^‐dependent CaMBDs (D.H. O'Day, unpublished data). Rab11A has one CaMBD (69TAGQERYRAITSAYYRGAVGALLV92) that possesses multiple binding motifs (1–8‐14, two 1–5‐10, 1–16, 1–14, 1–10) while Rab11C has a shorter CaMBD (68GQERFRAVTSGYYRGAVGAMI88) within which three motifs (1–10, 1–5‐10, 1–14) are evident. Drainin also contains a short Ca^2+^‐dependent CaMBD (380RTALSILRYFIS391) but it lacks any canonical motifs. Rab8A, on the other hand, reveals a putative CaMBD that lacks canonical motifs (71TAGQERFRTITTAYYRGAMGI91).

**Figure 2:**
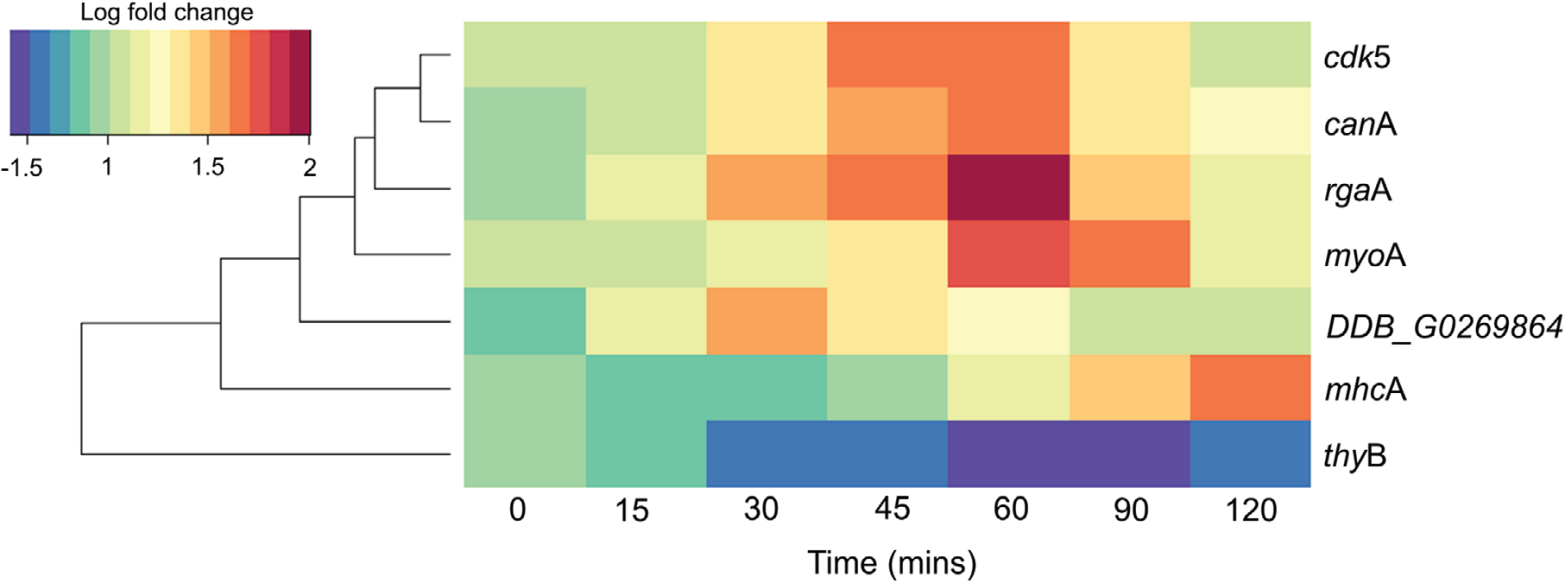
Calmodulin‐binding proteins are differentially regulated under hypertonic stress. A transcriptomic approach by Na *et al*. (2007) revealed genes that are differentially expressed at various time points (0–120 min) during hypertonic stress. Gene enrichment analysis of the 809 genes through the Gene Ontology (GO) database revealed an enrichment of calmodulin binding proteins (CaMBPs) (GO: 0005516). A binomial test was used to determine the enriched genes on the GO database. Seven of the identified genes encoded CaMBPs (listed to the right of the heat map); these are shown in the heat map with the associated log‐fold changes at various time points. A dendrogram (left of the heat map) describes the similarity in the transcriptional changes at various time points.

Na, Tunggal, & Eichinger (2007) examined the transcriptional changes during hypertonic stress in *D. discoideum* and identified 809 genes that were differentially expressed. Utilizing the Gene Ontology (GO) database, we identified an enrichment (binomial test) of genes encoding CaMBPs (GO: 0005516): *rgaA*, *mhcA*, *myoA*, *cdk5*, *thyB*, *canA*, and uncharacterized gene *DDB_G0269864* (Fig. 2). Two of the seven genes are linked to the cytoskeleton, encoding the myosin IA heavy chain (*myoA*) and the myosin‐2 heavy chain (*mhcA*). Cytoskeletal changes alter cell shape during hypertonic stress (where cells shrink and become rounded) and strengthen the cell cortex (Kuwayama *et al.,* 1996). Therefore, these two cytoskeletal CaMBPs are potentially regulated by CaM during the remodelling of the cytoskeleton during hypertonic stress.

CanA and Cdk5 are two well‐studied CaMBPs in *D. discoideum* (Dammann *et al.,* 1996; Huber & O'Day, 2011b, 2012b; Huber *et al.,* 2013). They also show time‐dependent co‐expression during hypertonic stress (Fig. 2). These transcriptional changes imply roles for these CaMBPs in osmoregulation. There is experimental evidence for this function for CanA, as reductions in its expression (through RNA interference) causes *D. discoideum* cells to be sensitive to cation‐induced hypertonic stress (Thewes *et al.,* 2016).

In response to hypertonic stress, *D. discoideum* cells secrete both cyclic GMP (cGMP) and H^+^ (Oyama, 1996; Oyama & Kubota, 1997). It is possible that this secretion is regulated by CanA and Cdk5. In human neurons, the combined activities of calcineurin and CDK5 regulate the exocytosis of neurotransmitters (Kim & Ryan, 2013). Interestingly, in *D. discoideum*, Cdk5 influences protein secretion (Sharma *et al.,* 2002). By contrast, CanA is required for the secretion of Ca^2+^ *via* its regulation of PatA (Moniakis, Coukell, & Janiec, 1999). Therefore, the actions of these known and potential CaMBPs may be key in regulating exocytosis during the hypertonic stress response in *D. discoideum*.

Mercanti *et al*. (2006) reported on a *D. discoideum* protein called Rh50 (Rhesus‐like glycoprotein, encoded by the *rhgA* gene) that is targeted to the CV system by a sequence of acidic residues (DDEEE) in the C‐terminal of the protein. Immunolocalization studies revealed that Rh50 colocalized with CaM at the membranes of the CV system but was not involved in osmoregulation. Uniprot lists two *D. discoideum* Rhesus‐like transmembrane glycoproteins, A and B, each of which possesses a single CaMBD in the cytoplasmic tail of the protein. The putative CaMBD for RhgA (425VILLQLKKIKGLKSKEY442) has a 1–10 binding motif while the RhgB CaMBD (431ILKALKKVGGLKAKQYY447) has 1–10, 1–12 and 1–5‐10 motifs. If both the CaM binding of these resident transmembrane proteins and their proposed role in ammonium transport can be validated, this would greatly advance our understanding of CV system function in *D. discoideum*.

## IX. CHEMOTAXIS AND AGGREGATION

*D. discoideum* cells can undergo random motility as well as chemotactic and chemokinetic movement directed either by folic acid (see Section III) or cAMP (developmental phase). There is also evidence for chemotaxis towards gradients of Ca^2+^ and arachidonic acid (Schaloske *et al.,* 2007; Lusche *et al.,* 2012). Understanding the genetic mechanisms that regulate CaM‐dependent chemotaxis in *D. discoideum* provides a unique opportunity to define the role of CaM across species due to the conserved chemotactic machinery shared with mammalian neutrophils and other white blood cell types. Granulocytes are a category of white blood cells characterized by the presence of granules in their cytoplasm. The CaM inhibitors trifluoperazine and W‐7 strongly inhibit chemotactic behaviour in rabbit peritoneal granulocytes (Elferlink, Deierkauf, & Riemersma, 1982). Regulation of serotonin‐induced human and mouse eosinophil chemotaxis may also require CaM. Using specific inhibitors, disruption of phosphatidylinositol 3‐kinase (PI3K), protein kinase C (PKC) and CaM, but not G(αi)‐proteins, prevents eosinophil rolling and actin polymerization suggesting that cell migration is dependent on these signalling molecules (Kang *et al.,* 2013).

**Figure 3:**
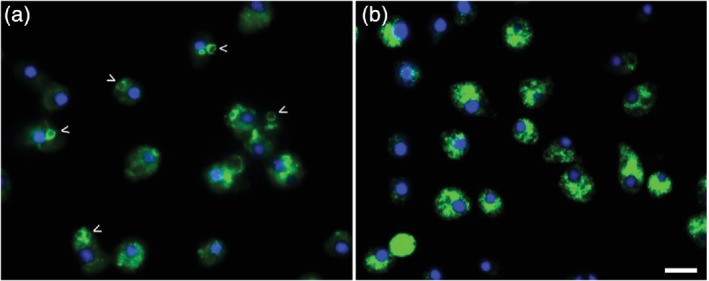
Mislocalization of calmodulin (CaM) in *htt*‐null cells. (A) Parental and (B) *htt*‐null cells were probed using mouse monoclonal anti‐CaM antibodies (clone IF11 Sigma) followed by goat anti‐mouse Alexa Fluor 488 secondary antibodies. Arrows denote CaM on the membranes of the CV system. CV system vacuoles are completely absent in mutant cells. DAPI (4′,6‐diamidino‐2‐phenylindole) was used to visualize nuclei (blue). Scale bar, 10  m. M.A. Myre, unpublished data).

*D. discoideum* as a model for chemotaxis continues to allow us to identify conserved genes not previously known to be involved in directed cell motility, and to test mutant cell lines for altered CaM‐binding kinetics, expression and intracellular localization. When starved on non‐nutrient agar plates, *D. discoideum* cells aggregate by chemotaxis in response to outwardly relayed, pulsatile waves of cAMP that are released from aggregation centres (Konijn *et al.,* 1967; Gerisch *et al.,* 1975). Myre *et al*. (2011) showed that cells deficient for orthologous huntingtin protein (HTT) were defective in chemotaxis in a spatial gradient of cAMP. Although mutations in the huntingtin gene due to poly‐Q expansion cause Huntington's disease (HD) in humans, the normal function of HTT remains obscure. Sequencing of the *D. discoideum* genome (Eichinger *et al.,* 2005) and many more invertebrate genomes presents an unprecedented opportunity to understand HTT function and its evolution. Suppression of HTT messenger RNA (mRNA) in *Ciona intestinalis* resulted in decreased motility; primary microglia from early postnatal HD mice are greatly impaired in their migration to chemotactic stimuli; compared to HTT(7Q/7Q) neuronal stem cells (NS), mutant Htt(140Q/140Q) and HTT knock‐out NS cells also showed significantly impaired motility (Kwan *et al.,* 2012; Ritch *et al.,* 2012; Idris, Thorndyke, & Brown, 2013). Interestingly, analysis of both human (3144 amino acids) and *D. discoideum* HTT (3095 amino acids) using the Calmodulin Target Database predicts a single CaM‐binding motif (1–8‐14) in each protein located toward the C‐terminal region of the protein (*D. discoideum* HTT, 1885LDLRKKQLLRLLSL1896; human HTT, 2538LKALDTRFGRKLSII2552). As discussed above (see Section VIII), CaM can be detected in the cytosol but is enriched on membranes of the CV system. *D. discoideum* cells that lack HTT under conditions of hypoosmotic stress die by rapid cell swelling followed by complete lysis within 3 h of exposure to water (Myre *et al.,* 2011). Importantly, metabolite abnormalities in HD brains might result from defects in osmoregulation (Patassini *et al.,* 2015). In addition, transient hyperosmotic treatment of human neuroblastoma SH‐SY5Y cell lines stably expressing mutant truncated huntingtin (Q88) significantly increases the number of cells with aggregates (Chun *et al.,* 2002). Curiously, microarray analysis of *D. discoideum* cells where the orthologous huntingtin gene has been knocked out by homologous recombination (*htt‐null*) identified a fivefold increase (*P* < 0.003) in the expression of *cmbB*; a sixfold decrease (*P* < 0.009) in *cmbC*; a fourfold decrease (*P* < 0.004) in *symA*, a CaMBP transporter protein; and a 2.5‐fold increase (*P* < 0.05) in CaM during chemotaxis (M.A. Myre, F.J.J. Chain & J.F. Gusella, unpublished data). Moreover, in parental cells, the immunolocalization of CaM can be seen in the cytosol and on the membranes of the CV system. However, in *htt*‐null cells CaM cannot be detected on the CV system and is found solely in the cytosol (Fig. 3) (M.A. Myre, A.L. Lumsden & J.F. Gusella, unpublished data). Taken together, these data suggest that the interdependence of CaM and HTT needs to be studied in more detail not only in *D. discoideum* but also in mammalian cells with relevance to HD.

Although the fine details of how divalent cations mediate the diversity of events underlying cell movement are still under analysis, Ca^2+^ signal transduction is essential for cAMP‐mediated chemotaxis in *D. discoideum* (e.g. Unterweger & Schlatterer, 1995; Schaloske *et al.,* 2007). The role of CaM in chemotaxis was demonstrated by Gauthier & O'Day (2001) who also revealed a diversity of CaMBPs, a group of which were linked to either folic acid‐ or cAMP‐mediated chemotaxis. CaM‐dependent protein phosphorylation coincided with these chemotactic events (Gauthier & O'Day, 2001). At the time, the specific proteins that were phosphorylated and the CaMBPs involved remained to be identified. More critically, chemotaxis is a complex cellular mechanism and the exact components that were CaM‐dependent were unknown.

Other potential CaMBPs have been shown to be involved in chemotaxis but their ability to bind CaM remains to be studied. Probably the best example of this is the IQGAP family of proteins. IQGAP proteins have been linked to chemotaxis and other life cycle events (Catalano & O'Day, 2008). Named for the presence of IQ motifs, four members of the IQGAP family exist in *D. discoideum*: DdIQGAP1 (RgaA), DdIQGAP2 (GapA) and DdIQGAP3 (IqgC) (Shannon, 2012). From yeast to mammals, IQGAP proteins mediate multiple cellular processes including cell adhesion, cytokinesis, exocytosis and cell motility. Like its mammalian counterparts, *D. discoideum* IQGAP1 (RgaA) has two putative IQ motifs (114IQELKRNLVSEVRR127; 193LQTEPKYLAGLVYL206) as well as a Ca^2+^‐dependent 1–16 motif (267MIITYNKRKQGTDYLKAVIG286; Catalano & O'Day, 2008). GapA and IqgC are essential for cytokinesis. All three proteins have critical but overlapping functions in chemotaxis apparently acting through their association with F‐actin and cortexillins I and II. The CaM‐binding ability of the IQ motifs has not been verified. Coupled with the absence of F‐actin binding domains in the proteins, this leaves many questions unanswered about these potential CaMBPs.

## X. CALCIUM‐MEDIATED CHEMOTAXIS

Scherer *et al*. (2010) revealed that steep gradients of Ca^2+^ could direct the positive chemotaxis of *D. discoideum* cells. The authors showed the gradients had to be extremely steep, contrasting with the actual levels of Ca^2+^ present during normal chemotaxis and cellular orientation in increasing spatial gradients of cAMP. In support of this, *htt*‐null cells failed to undergo both K^+^‐facilitated chemotaxis in spatial gradients of the major chemoattractant cAMP, and chemotaxis up a spatial gradient of Ca^2+^, but behaved normally in Ca^2+^‐facilitated cAMP‐mediated chemotaxis and Ca^2+^‐dependent flow‐directed motility (Wessels *et al.,* 2014). While the significance of this mode of chemotaxis requires further study, it is possible that rather than being a true form of chemotaxis, non‐biological high levels of Ca^2+^ may simply activate the downstream Ca^2+^/CaM‐mediated pathways normally associated with cAMP‐mediated chemotaxis. Any differences between cAMP‐mediated and Ca^2+^‐mediated chemotaxis could be due to the extremely high levels of Ca^2+^ activating non‐canonical events or other factors. For example, the requirement for the plasma membrane transporter IplA in Ca^2+^‐mediated but not cAMP‐mediated chemotaxis could simply be due to the inability of Ca^2+^ gradients to be effective in *iplA* mutants, which would not allow the uptake of Ca^2+^ into cells. Regardless, the role of CaM, a primary Ca^2+^ target, in Ca^2+^‐mediated chemotaxis was not evaluated.

## XI. BLEBBING MOTILITY

Under certain conditions, *D. discoideum* uses a blebbing‐based form of cell motility during chemotaxis (Langridge & Kay, 2006; Zatulovskiy *et al.,* 2014). Blebs are small membrane protrusions whose formation has been shown to be dependent on Ca^2+^, CaM, the CaMBP ADP‐ribosylation factor 6 (ARF6) and phospholipase C in other species (Yanase *et al.,* 2010). These signalling components regulate myosin II‐based contractility. The role of PI3K and myosin II in bleb formation was subsequently validated in *D. discoideum* (Zatulovskiy *et al.,* 2014). However, they dismissed the role of Ca^2+^ because *iplA* ^*−*^ cells did not show altered blebbing. This conclusion may have been premature since previous research has shown that *iplA* ^*−*^ cells still undergo altered but functional Ca^2+^ signalling during chemotaxis (Schaloske *et al.,* 2005; Lusche *et al.,* 2012). Thus, it will be critical for future work to resolve this apparently contradictory conclusion.

## XII. EXTRACELLULAR MATRIX AND SECRETED PROTEINS

In *D. discoideum*, CaM is detected extracellularly during all stages of the asexual life cycle where it is thought to function as a modulator of growth, cAMP‐mediated chemotaxis, and proteolysis through its interaction with specific CaMBPs (Suarez *et al.,* 2011; O'Day *et al.,* 2012). The functions of eCaM during growth were discussed above (see Section V). Bakthavatsalam & Gomer (2010) used mass spectrometry to reveal CaM, as well as several putative and established CaMBPs including Cad1, puromycin‐sensitive aminopeptidase B (PsaB), Cbp4a, and CmbC, in conditioned buffer harvested from parental AX2 cells undergoing multicellular development. However, none of these proteins was discussed or studied further (Bakthavatsalam & Gomer, 2010). Huber (2017) used a modified protocol to confirm the secretion of CaM during the early stages of multicellular development of AX3 cells, another parental strain commonly used in *D. discoideum* research. In addition to Cad1 and PsaB, the latter work also revealed the presence of the CaMBPs PsaA, PgkA and RgaA in conditioned starvation buffer. Together, these results validate the secretion of CaM and CaMBPs during *D. discoideum* multicellular development.

Once secreted, proteins can either diffuse away, bind to the cell surface to modulate cellular processes, or be incorporated into the ECM (Huber & O'Day, 2017). The cells that comprise the *D. discoideum* slug are surrounded by a slime sheath that functions as a primitive ECM in the organism. Huber & O'Day (2015) used mass spectrometry to reveal the proteins contained within the sheath ECM of two wild‐type strains of *D. discoideum*, NC4 and WS380B. Several putative and established CaMBPs were detected in the slime sheath of both strains including Cad1, Ca^2+^‐dependent cell adhesion protein 2 (Cad2), MhcA (myosin heavy chain A), Cbp4b, and Ca^2+^‐binding protein G (CbpG) (Huber & O'Day, 2015). In addition, RgaA, PsaB, and CmbC were detected in NC4 ECM, while PsaA, CbpA (CBP1), and Ca^2+^‐dependent cell‐to‐cell adhesion protein 3 (Cad3) were detected in WS380B ECM. While proteomics‐based analyses and western blotting support the secretion of CaM during multicellular development, and there is some evidence from immunolocalization experiments to support the presence of CaM in the ECM, mass spectroscopy did not detect CaM in the sheath ECM (Bakthavatsalam & Gomer, 2010; Suarez *et al.,* 2011; O'Day *et al.,* 2012; Huber & O'Day, 2015; Huber, 2017). These observations indicate that CaM might not reside long within the ECM, may be retained but undetectable due to its binding to CaMBPs, or may be present at levels that are too low for immunolocalization. They may also reflect the fact that the CaM secretion studies were performed using parental axenic strains of *D. discoideum* (AX2, AX3), while the sheath ECM proteomics study was performed using wild‐type strains isolated from nature (NC4, WS380B). Nonetheless, these results suggest that CaMBPs function in the ECM to regulate cellular events during the mid‐to‐late stages of *D. discoideum* development.

## XIII. A MATRICELLULAR CAMBP THAT REGULATES CELL MOTILITY

The best characterized extracellular CaMBP in *D. discoideum* is CyrA, which was first isolated using the CaM‐binding overlay technique (CaMBOT; O'Day, 2003; O'Day & Huber, 2013). CyrA contains an identified CaMBD and binds to CaM in both the presence and absence of Ca^2+^ (Suarez *et al.,* 2011; Huber, Suarez, & O'Day, 2012). Prior to being secreted, CyrA localizes to the ER, particularly its perinuclear component (Huber *et al.,* 2012). Secretion of CyrA requires the release of intracellular Ca^2+^ as well as active CaM, PI3K and phospholipase A2 (PLA2). While CyrA is secreted throughout the *D. discoideum* life cycle, secretion is highest during the slug stage of multicellular development where it localizes to the slug sheath ECM (Suarez *et al.,* 2011; Huber *et al.,* 2012).

A body of work has established CyrA as the first matricellular protein identified in a eukaryotic microbe. Matricellular proteins localize to the ECM and contain binding sites for both the ECM and cell surface, however they do not directly contribute to the organization or physical properties of the ECM (Murphy‐Ullrich & Sage, 2014). Matricellular proteins are also expressed at high levels during development, associate with extracellular proteases and growth factors, and modulate cellular processes by binding to the cell surface and initiating intracellular signal transduction (Murphy‐Ullrich & Sage, 2014). As will be discussed below, CyrA has all these attributes, which allows it to be classified as a matricellular protein in *D. discoideum*.

During growth and development, full‐length CyrA (63 kDa) is cleaved into two major C‐terminal products of molecular weights 45 kDa and 40 kDa (Suarez *et al.,* 2011; Huber *et al.,* 2012). Cleavage of CyrA is developmentally regulated and modulated by CaM, which protects CyrA from proteolysis (Suarez *et al.,* 2011). CyrA also contains four tandem EGF‐like repeats in its C‐terminus, which are present in both CyrA cleavage products. In mammalian matricellular proteins, EGF‐like repeats modulate cell motility by binding to the cell surface (Iyer *et al.,* 2008). Interestingly, a synthetic peptide (DdEGFL1), equivalent in sequence to the first 18 amino acids of the first EGF‐like repeat of CyrA, functions extracellularly to increase both random cell motility and cAMP‐mediated chemotaxis in a variety of wild‐type and parental strains of *D. discoideum* (e.g. NC4, AX2, AX3, KAX3, DH1) (Huber & O'Day, 2009, 2011a, 2011b; Suarez *et al.,* 2011; Nikolaeva, Huber, & O'Day, 2012; Huber & O'Day, 2012a). The role of CyrA in the regulation of cell motility was validated by showing that overexpression of the protein increases cAMP‐mediated chemotaxis (Huber *et al.,* 2012). Unlike cAMP, DdEGFL1 does not function as a chemoattractant during the early stages of multicellular development (Nikolaeva *et al.,* 2012). However, the peptide has been detected on the membranes of cells capped with concanavalin A suggesting that a receptor exists for EGF‐like repeats in *D. discoideum* (Huber *et al.,* 2012). Furthermore, the dose‐dependent enhancement of motility by DdEGFL1 increases as cells are starved for longer periods of time suggesting that starvation increases the expression or presentation of a receptor that binds EGF‐like repeats (Suarez *et al.,* 2011). Finally, treatment of cells with DdEGFL1 inhibits CyrA proteolysis suggesting that this domain plays a key role in regulating the cleavage of CyrA *via* a negative feedback mechanism (Suarez *et al.,* 2011).

DdEGFL1 functions independently of the pathway regulating the response of cells to cAMP, since the peptide increases the movement of cells lacking the cAMP receptors CarA and CarC, as well as cells that lack the G‐proteins coupled to these receptors (Huber & O'Day, 2011a). Instead, the peptide activates a novel pathway involving Ca^2+^, CaM, RasG, PI3K, and PLA2, which leads to increased amounts of actin and myosin II heavy chain in the cytoskeleton (Huber & O'Day, 2009, 2011a, 2013). DdEGFL1 also sustains the threonine phosphorylation of the cytoskeletal protein vinculin B (VinB) during the early stages of *D. discoideum* development (Huber & O'Day, 2009, 2012a). In addition, the 45 kDa CyrA cleavage product has been detected in VinB immunoprecipitates, thus providing a link between the increased cell motility and a specific cytoskeletal component. Finally, the cytoskeletal proteins talin B and paxillin B, as well as tyrosine kinase activity mediated by protein kinase A (PKA) signalling, are also involved in translating the DdEGFL1 signal into an increase in cell motility (Huber & O'Day, 2012a). In total, these findings show that CyrA, and possibly other secreted CaMBPs, play an important role in regulating cell motility in *D. discoideum*.

## XIV. CELL DIFFERENTIATION AND MORPHOGENESIS

**Figure 4:**
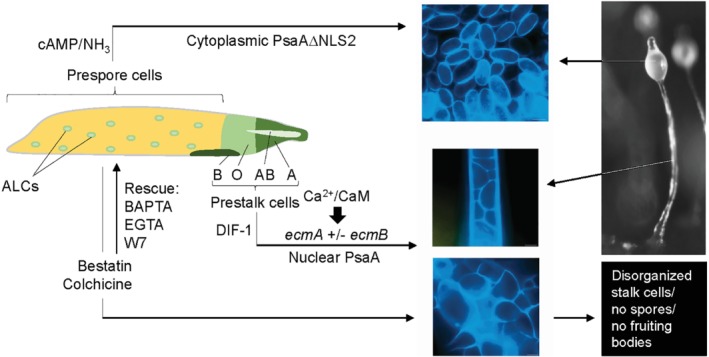
Calmodulin and calmodulin‐binding proteins linked to stalk cell and spore differentiation in *Dictyostelium discoideum*. The stalk cells and spores were stained with Calcofluor. ALC, anterior‐like cell; BAPTA, 1,2‐Bis(2‐aminophenoxy)ethane‐N,N,N′,N′‐tetraacetic acid tetrakis(acetoxymethyl ester); CaM, calmodulin; cAMP, cyclic AMP; DIF‐1, differentiation inducing factor‐1; EGTA, ethylene glycol‐bis(β‐aminoethyl ether)‐N,N,N′,N′‐tetraacetic acid; PsaA, puromycin‐sensitive aminopeptidase; W‐7, N‐(6‐aminohexyl)‐5‐chloro‐1‐naphthalenesulfonamide.

In *D. discoideum* (see Section I), multicellular morphogenesis and differentiation generates a fruiting body consisting of a mass of viable spores supported by a stalk of dead cells. During the multicellular slug stage, the prestalk cells A, AB and O (pstA, pstB, pstO) mainly reside at the front of the slug and prespore cells at the rear (Fig. 4). A number of ALCs are also localized within this prespore region. The pstA, pstAB and pstO cells are defined by their unique patterns of expression of certain genes (e.g. *ecmA*, *ecmB*). The importance of Ca^2+^, *ecmB* and the regulatory role of differentiation inducing factor‐1 (DIF‐1) in stalk cell differentiation has been well documented. The endogenous morphogen DIF‐1 and specific pharmacological treatments induce *ecmB* expression and stalk cell differentiation *in vitro* through the elevation of intracellular Ca^2+^ levels (e.g. Williams *et al.,* 1987; Kubohara & Okamoto, 1994; Kay, Flatman, & Thompson, 1999; Kubohara *et al.,* 2007; Saito, Kato, & Kay, 2008). *In vivo* intracellular Ca^2+^ levels are significantly higher in prestalk *versus* prespore cells and the elevation of Ca^2+^ levels generates slugs with increased prestalk zones that produce stalky fruiting bodies that are deficient in spores (Abe & Maeda, 1989; Baskar *et al.,* 2000). An early pharmacological study showed that CaM antagonism inhibited Ca^2+^‐induced *ecmB* expression *in vitro* (Verkerke‐van Wijk *et al.,* 1998). Poloz & O'Day (2010) followed this up by showing that Ca^2+^ and CaM regulate *ecmB* expression as well as pstAB/pstB cell differentiation in normal slugs *in vivo*. Furthermore, Ca^2+^‐based pharmacological treatments had differential effects on *ecmB* expression and cell differentiation in the anterior *versus* posterior zones of slugs. For example, increased intracellular levels of Ca^2+^ increased the number of *ecmB*‐expressing cells in the anterior region of slugs, while decreasing intracellular Ca^2+^ levels or antagonizing CaM led to an increase in the number of *ecmB*‐expressing cells in the posterior.

The central role of Ca^2+^/CaM signalling in stalk cell differentiation was further emphasized by results that came from an unexpected direction. For almost half a century, it has been known that colchicine induces developmental defects in *Hydra*, *Drosophila melanogaster*, *Xenopus laevis* and other species, typically altering the pattern and polarity of the whole organism or of specific structures (reviewed in O'Day & Durston, O'Day & Durston, 1978; Poloz & O'Day, 2012). Similarly, in *D. discoideum*, colchicine inhibits slug formation while inducing disorganized mounds of stalk cells and inhibiting spore formation (O'Day & Durston, O'Day & Durston, 1978). The developmental effects were identified by the altered pattern of neutral red staining, a vital‐dye prestalk marker in *D. discoideum*. Poloz & O'Day *et al*. (2012) revealed that, contrary to the expectations of previous researchers, the effects of colchicine were not due to the disruption of microtubules but rather were caused by an increase in intracellular Ca^2+^ levels that in turn activated CaM. They then showed in greater detail that colchicine affects cell motility, disrupts morphogenesis, inhibits spore cell differentiation and induces stalk cell differentiation through a Ca^2+^ and CaM‐dependent signal transduction pathway.

In keeping with this, colchicine‐treated cells could be rescued by lowering intracellular Ca^2+^ levels with BAPTA‐AM (1,2‐Bis(2‐aminophenoxy)ethane‐N,N,N′,N′‐tetraacetic acid tetrakis(acetoxymethyl ester)) or extracellular levels with EGTA (ethylene glycol‐bis(β‐aminoethyl ether)‐N,N,N′,N′‐tetraacetic acid) or by antagonizing CaM using W‐7 (Fig. 4). Rescued cells could complete development to form fruiting bodies with normal stalks and viable spores. Colchicine also induced the expression of stalk‐specific *ecmB* in both wild‐type cells and a DIF‐1 (*dmtA* ^*−*^) mutant, revealing that this induction was DIF‐1 independent. In addition, colchicine inhibited the expression of spore‐specific genes (*cotB*, *spiA*), fitting with the absence of spores in colchicine‐treated cultures. Since stalk cell differentiation bears similarities to ACD, colchicine‐induced stalk cell formation could be useful for autophagy research in *D. discoideum* (Chen & Schapp, Chen & Schaap, 2012; Schaap, 2016).

While CaM primarily functions during stalk differentiation, there is an indirect relationship between certain CaMBPs and spore differentiation. As discussed above (see Section IV), the cell‐cycle‐linked nuclear/nucleolar CaMBP NumA1 binds to various proteins. *D. discoideum* PsaA is mainly a nuclear protein that associates with NumA1 in the nucleoplasm, but not the nucleolus, during interphase (Catalano *et al.,* 2011). The enzyme is also detectable at low levels in the cytoplasm. Overexpression of full‐length PsaA inhibits spore differentiation leading to increased stalk cell formation. By contrast, deletion of a critical NLS resulted in cytoplasmic localization but, as predicted, no nuclear localization. This altered locational distribution resulted in the differentiation of spores and the absence of stalk cells (Fig. 4). By contrast, the inhibition of aminopeptidase activity with bestatin had a similar effect as treatment of slugs with colchicine, inducing cells to differentiate into stalk cells. Bestatin methyl ester (BME) is an inhibitor of Zn^2+^‐binding aminopeptidases (see Poloz, Catalano, & O'Day, 2012).

Cad1 displays differential localization, being predominantly a cell surface protein in prestalk cells but localizing internally in prespore cells (Sriskanthadevan *et al.,* 2011). Cad1 is transported and inserted into the cell surface *via* the CV system. Experiments with chimeras of parental and knockout (*cadA* ^*−*^) cells revealed that Cad1 fulfils a primary function in cell sorting. Although Cad1 is a proven CaMBP as discussed above (see Section VIII), the role of CaM in the Cad1‐mediated events discussed here was not investigated.

## XV. PSEUDOPLASMODIUM REGENERATION

The regeneration of any multicellular eukaryotic structure involves some basic developmental events: wound healing, cell de‐differentiation (loss of cell‐type specific characteristics), re‐establishment of pattern and polarity, and cell re‐differentiation (specialization of cells and tissues) (Alibardi, 2017; Wells & Watt, 2018). The existence of stem cells can serve as a resupply unit of multipotent, pluripotent or totipotent cells that can supplement the population of de‐differentiated cells. While *D. discoideum* appears to lack 'stem cells', it does carry out the other regenerative events and processes: wound healing, de‐differentiation, and re‐differentiation with the re‐establishment of a normal pattern and polarity. That said, currently the regeneration of pseudoplasmodia is one of the least explored areas of research in *D. discoideum*.

Extensive early research into *D. discoideum* regeneration was designed to determine if the pattern and polarity of the multicellular slug was due to cell differentiation or sorting of prespore (rear) and prestalk (front) cells (Bonner, 1951). Along with accumulated evidence and based on cutting and grafting experiments by Francis & O'Day (Francis & O'Day, 1971), it was argued that cell differentiation/re‐differentiation as well as cell sorting were at play, thus fitting with models of animal development and regeneration, which extends to early metazoans like sponges. The results of these regeneration experiments fit with the argument that re‐differentiation must occur in *D. discoideum*, while the presence of various subpopulations of developing cell types indicates that at least some cell sorting occurs.

As shown in Fig. 4, the front of the slug is inhabited by future stalk cells while the rear is primarily comprised of prespore cells. If the tip is cut off and the slug is forced to culminate immediately, then a disproportional fruiting body is formed from each segment. The front will be stalky with a small spore mass, while the rear will have a thin stalk with an enlarged spore mass. However, letting the cut pieces migrate before fruiting allows the normal proportioning to be regenerated. These events coincide with cell‐specific gene expression patterns within each segment that are dependent on CaM function.

Ca^2+^/CaM regulate *ecmB* expression as well as pstAB/pstB cell differentiation in regenerating slugs (Poloz & O'Day, 2010). During regeneration, treatments that decrease intracellular Ca^2+^ or antagonize CaM also decrease *ecmB* expression and the differentiation of pstAB/pstB cells. The accumulated evidence suggests that CaM has no effect on slug migration but does function in the resultant morphology of regenerating slugs. CaM inhibition generates long thin slugs regenerated from the prespore end and short, wider front‐end slugs (Poloz & O'Day, 2010). However, the reasons for these morphological differences remain to be studied. CaM was also shown to function in other aspects of regeneration such as the rate of recovery of and cell motility in regenerating pseudoplasmodia. Understanding the biomolecular events and the role of CaM in *D. discoideum* regeneration could provide valuable evolutionary insights into this process.

## XVI. SPORE GERMINATION

In *D. discoideum*, spore germination begins a new life cycle. In nature, the dormant spores become activated by the presence of an appropriate microbial food source. In the laboratory, they can be induced to germinate through the addition of an endogenous autoactivator or by the controlled application of heat (Cotter & Raper, 1966; Cotter *et al.,* 1992). Spore germination consists of four stages: activation, lag, swelling and amoeba emergence. Three of these are active transition phases (activation, swelling, emergence) characterized by specific transcriptional events (Xu *et al.,* 2004). CaM antagonists inhibit autoactivation but not heat‐induced activation, however both types of induced swelling and emergence are CaM dependent (Lydan & Cotter, 1994; Lydan, Cotter, & O'Day, 1994a, 1994b). While a diverse population of Ca^2+^‐dependent and ‐independent CaMBPs are present during both types of induced germination, a single unidentified Ca^2+^‐dependent CaMBP of 64 kDa (possibly CyrA) was linked specifically to autoactivated germination (Lydan *et al.,* 1994a). In addition, emergence was characterized by the loss of a 52 kDa Ca^2+^‐independent CaMBP. Despite numerous changes in phosphoproteins specifically associated with either type of activation, they could not be linked to the activity of a CaM‐dependent kinase (Lydan *et al.,* 1994b).

As discussed above, IplA is a CaMBP involved in blebbing motility (see Sections VII and XI). Interestingly, inositol 1,4,5‐trisphosphate, the activating substrate for IplA, also causes the autoactivation of spores (Lydan & Cotter, 1994), whereas inhibitors of Ca^2+^ efflux from intracellular stores inhibit autoactivation. Another phenotype observed in autophagy mutants was the lack of spores, suggesting defective physiological processes that control spore formation and dormancy (Otto *et al.,* 2003). This may be due to a lack of autophagic vacuoles found in dormant spores and during the germination process (Cotter *et al.,* 1992). Increased levels of intracellular Ca^2+^ promote the actions of the CaM‐dependent kinases (CaMKs) that are involved in the initiation of autophagy (Høyer‐Hansen *et al.,* 2007; Pfisterer *et al.,* 2011; Evankovich *et al.,* 2012). These include CaMKs I, II and IV which act to promote autophagy and autophagosome formation in human cells (Pfisterer *et al.,* 2011; Evankovich *et al.,* 2012; Li *et al.,* 2017). In addition, CaM‐dependent phosphorylation does occur in response to starvation in *D. discoideum*, which has orthologs of CaMKI and CaMKII (Gauthier & O'Day, 2001; Goldberg *et al.,* 2006). Although CaM‐dependent phosphorylation overlaps with starvation in *D. discoideum*, more work in this area is required to discern its role in autophagy and spore germination.

**Figure 5:**
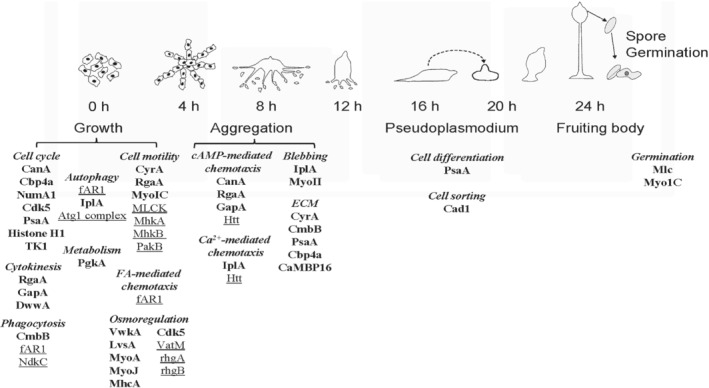
Calmodulin‐binding proteins (CaMBPs) linked to specific events during asexual development in *Dictyostelium discoideum*. Names in bold indicate experimentally proven CaMBPs; underlined names are putative CaMBPs containing calmodulin binding domains (CaMBDs). Atg1 complex, autophagy‐related 1 complex; Cad1, Ca^2+^‐dependent, cell‐to‐cell adhesion molecule 1; CaMBP16, calmodulin binding protein 16; cAMP, cyclic 3′,5′ adenosine monophosphate; CanA, calcineurin A; Cbp‐4a, calcium‐binding protein 4a; Cdk5, cyclin‐dependent kinase 5; CmbB, calmodulin‐binding protein 46; CyrA, cysteine‐rich protein A; DwwA, *Dictyostelium* WW domain‐containing protein A; ECM, extracellular matrix; FA, folic acid; fAR1, colic acid receptor 1; GapA, IQGAP family member DdIQGAP2; H1, Histone H1; Htt, huntingtin protein; IplA, inositol 1,4,5‐trisphosphate receptor, LvsA, BEACH domain‐containing protein; MhkA, myosin heavy chain kinase A; MhkB, myosin heavy chain kinase B; Mlc, myosin light chain; MLCK, myosin light chain kinase; MlhcA, myosin‐2 heavy chain; MyoIC, myosin‐1C; MyoII, myosin II; MyoA, myosin A; MyoJ, myosin J; NdkC, nucleotide diphosphate kinase C; NumA1, nucleomorphin A1; PakB, p21‐activated protein kinase; PAT1, Ca^2+^‐ATPase; PgkA, phosphoglycerate kinase; PsaA, puromycin‐sensitive aminopeptidase; RgaA, ras GTPase‐activating‐like protein A; rhgA, Rhesus‐like glycoprotein A; rhgB, Rhesus‐like glycoprotein B; TK1, thymidine kinase; VatM, V‐ATPase subunit M; VwkA, Von Willebrand factor kinase A.

A histidine kinase (DhkB) was shown to regulate spore germination but its ability to phosphorylate other proteins was not studied nor is there any evidence this kinase is activated by CaM in other species (Zinda & Singleton, 1998). Previous work performed a transcriptomics analysis of heat shock and dimethyl sulfoxide‐induced spore germination to monitor changes in global transcription patterns (Xu *et al.,* 2004). A cursory analysis of their raw data reveals the differential expression of CaM and a number of verified and putative CaMBPs including CaMBP38, CmbB, CmbC and Myo1C (Fig. 5). In addition, CaMKI and a non‐specified Ca^2+^/CaM‐dependent protein kinase were also expressed. While CanA plays a role in spore differentiation as detailed above (see Sections III & VIII), its role in spore germination has not been studied nor was its presence detected in the transcriptional analysis (Horn & Gross, 1996; Xu *et al.,* 2004). Clearly a detailed analysis of the data of Xu *et al*. (2004) in relation to the function of CaM during the different stages of germination could be enlightening. Despite an enthusiastic start into the role of CaM in spore germination, there has been no apparent subsequent research into this topic.

## XVII. CONCLUSIONS

1. CaM carries out critical functions for diverse events during the asexual development of *D. discoideum* (Fig. 1). Coupled with this, specific CaMBPs have been identified that are either linked with or critical to many of those events (Fig. 5). This makes *D. discoideum* one of the most comprehensively studied eukaryotes in terms of Ca^2+^ signalling and CaM function.
2. While not detailed here, putative and experimentally defined CaMBDs of multiple other CaMBPs have been identified (Catalano & O'Day, 2008). To date, the CaMBDs that have been identified primarily fall into canonical motif subclasses. Only a few have been shown to be non‐canonical motifs. Does this suggest some evolutionary distinction in CaM‐binding across species? Did canonical CaMBDs appear first during evolution?
3. A perplexing aspect of CaM function in *D. discoideum* is the lack of a large number of Ca^2+^/CaM‐dependent kinases. In mammals, it has long been known that there is a critical interplay between CanA dephosphorylation and Ca^2+^/CaM‐dependent kinase phosphorylation of specific proteins (e.g. Levitan, 1994; Penny & Gold, 2018). *D. discoideum* does have a well characterized CaM‐dependent CanA (Dammann *et al.,* 1996). However, a CaM‐dependent phosphorylation–dephosphorylation interplay has not been identified in *D. discoideum* in spite of the presence of multiple CaMKs. The 21 CAMK group members consist of both potential Ca^2+^/CaM‐activated kinase or CAMK1 family members (eight genes) and CaM‐independent kinases (e.g. CAMK‐like RAD53) (Goldberg *et al.,* 2006). An enzyme identified originally as *D. discoideum* myosin light chain kinase (MLCK) is in fact a CaMK1 family member. To date, only the alpha kinase VwkA has been proved to be activated by Ca^2+^/CaM which is confusing considering the evolutionary consistency of multiple Ca^2+^/CaM‐dependent kinases coupled with the central role of CaM in the life cycle of *D. discoideum*.
4. There is no doubt that CaM is one of the most perplexing of all regulatory proteins. Despite its central role in a multitude of basic biological processes, the way it binds to its Ca^2+^‐dependent target proteins is still a comparatively poorly understood process. While multiple pharmacological CaM antagonists exist, none target specific CaMBPs. What will be key in the future is to develop anti‐CaM agents that affect its binding to specific CaMBPs. Already new antagonists have been designed (e.g. Audran *et al.,* 2013). CaM‐ and CaMBP‐specific peptides also are under development. For example, a peptide sequence generated from CaM disrupts its binding to huntingtin reducing its cytotoxicity in differentiated neuroblastoma cells (SH‐SY5Y) and providing neuroprotection in a mouse (R6/2) model (Dudek, Dai, & Muma, 2008, 2010).
5. The ease of working with *D. discoideum* and its genetic tractability suggests it could be on the forefront of biomedical research on CaM. For example, *D. discoideum* has become a powerful model for gathering new insights into proteins linked to Batten disease [the neuronal ceroid lipofuscinoses (NCLs); McLaren, Mathavarajah, & Huber, 2019]. The NCLs are a group of lysosomal storage disorders that are the most common form of neurodegeneration in children. Recent work showed that 11 of the 13 human NCL proteins contain putative CaMBDs (Mathavarajah, McLaren, & Huber, 2018a). The two Batten disease proteins that do not contain putative CaMBDs are potentially linked to CaM through their association with cathepsin L, which contains a putative CaMBD (Mathavarajah *et al.,* 2018a). Since *D. discoideum* is an established NCL model, this presents an opportunity in the future to study the relationship between CaM and the NCL proteins. Together, these observations highlight the need to study CaM and its role in human disease.
6. CaM is central to the Ca^2+^ signalling events that mediate a diverse number of cellular and developmental events in this model eukaryote and, hopefully, future studies will take that one step further to find out how it performs its various jobs. While questions remain, the universal importance of CaM and its binding proteins in all aspects of the life cycle of *D. discoideum* is firmly established and worthy of more intense analysis.
7. A recent report has detailed the events involved in CaM binding providing insight into the promiscuous binding mechanism that defines this critical regulatory protein (Westerlund & Delemotte, 2018). In addition, Li *et al*. (2018) recently defined a short‐linear motif (SLM) feature for predicting CaMBDs. An extensive and detailed review of phospho‐calmodulin function in pathophysiological processes (Villalobo, 2018) has illuminated how post‐translational modifications can alter the function of this essential protein and how it binds to its CaMBPs.
